# Site-Specific Changes in Cytosine Methylation in Promoters of the Genes Encoding the Membrane Subunits of Succinate Dehydrogenase During Germination of Maize Seeds

**DOI:** 10.3390/ijms26168010

**Published:** 2025-08-19

**Authors:** Dmitry N. Fedorin, Alexander T. Eprintsev, Abir U. Igamberdiev

**Affiliations:** 1 Department of Biochemistry and Cell Physiology, Voronezh State University, 394018 Voronezh, Russia; rybolov@mail.ru (D.N.F.); bc366@bio.vsu.ru (A.T.E.); 2 Department of Biology, Memorial University of Newfoundland, St. John’s, NL A1C 5S7, Canada

**Keywords:** maize (*Zea mays* L.), succinate dehydrogenase, promoter methylation, bisulfite sequencing

## Abstract

The cytosine methylation status of symmetric and asymmetric sites of promoters of the genes encoding the membrane-bound subunits C and D of succinate dehydrogenase (SDH) was assessed during the germination of maize (*Zea mays* L.) seeds, when the stored lipids were utilized and the glyoxylate cycle produced succinate. The results of bisulfite sequencing of the promoters of *Sdh* genes in maize scutella allowed us to determine the cytosine methylation status in the CG, CNG, and CNN sites. The observed site-specific changes in the cytosine methylation status of the *Sdh3-1* and *Sdh3-2* genes encoding the SDH subunit C indicate an important role in controlling their transcriptional activity. In contrast, no marked changes were observed in the methylation of promoters of the *Sdh4* gene, encoding SDH subunit D. The analysis of changes in the activity of the CG, CNG, and CNN DNA methyltransferases revealed the redistribution of activity between CG, CNG, and CNN DNA methyltransferases toward an increase in the proportion of CG DNA methyltransferases. The locus-specific methylation dynamics of SDH membrane subunit promoters during maize germination have been demonstrated. It is concluded that the changes in the cytosine methylation status may play a role in the regulation of the expression of the *Sdh* genes in the course of the conversion of succinate formed in the glyoxylate cycle.

## 1. Introduction

Succinate dehydrogenase (SDH; EC 1.3.99.1) is embedded in the inner mitochondrial membrane, representing both complex II of the mitochondrial electron transport chain (ETC) and a functional part of the tricarboxylic acid (TCA) cycle. The enzyme consists of four subunits: two hydrophilic proteins of the mitochondrial matrix (SDHA and SDHB) and two hydrophobic membrane-bound proteins (SDHC and SDHD) [1,2,3]. For plants, up to four additional plant-specific subunits were demonstrated [4,5,6]. The presence of a family of *Sdh* genes has been shown, which includes the *Sdh3-1* and *Sdh3-2* genes with different chromosomal localizations, encoding the membrane-bound cytochrome *b*-containing subunit C, and the only gene *Sdh4* encoding another hydrophobic subunit D [2,3].

The polymorphism of the genes of the SDH complex in plants requires the operation of the system of regulation and coordination of their transcriptional activity to control the synthesis of peptides of the corresponding subunits. Previously, the differential expression of *Sdh* genes in scutella during the germination of maize seeds was demonstrated [7,8]. Revealing the details of this mechanism is important for understanding the regulation of physiological processes in plants during seed germination and in response to environmental factors [9]. In the oil-storing tissues of germinating seeds, such as the scutella of cereals and the cotyledons of many dicots, the glyoxylate cycle is operating, which produces high amounts of succinate utilized in mitochondria by SDH. The section of the TCA cycle utilizing succinate is induced to a high degree during this period [10]. The possibility of the conversion of the excess of succinate in glyoxysomes by a membrane-bound oxidase, with low affinity to succinate, has been shown [11]. It was demonstrated that isocitrate lyase, the enzyme producing succinate in the glyoxylate cycle, controls the subsequent metabolic processes that lead to the formation of sugars via gluconeogenesis [12]. This control occurs at different levels, one of them being the epigenetic mechanism of cytosine methylation. This was demonstrated, in particular, for the regulation of SDH subunits [7,13,14].

DNA methylation is an important modification for gene regulation and genomic DNA stability in eukaryotes [15,16]. This epigenetic process is part of the transcriptional regulation of genes, since changes in its patterns can lead to developmental abnormalities in organisms. In plants, DNA methylation is regulated by a complex interaction between several methylating and demethylating enzymes [17]. The differences in the regulation of methylation of different cytosine sites indicate the multidirectional control of the transcriptional activity of target genes, including *Sdh*. An important point is the study of gene regulation, in particular relating to changes in the cytosine methylation status in symmetric (CG and CNG, where N = A, C, or T) and asymmetric (CNN) sites that provide the overall methylation level of the regulatory regions of genes. Cytosine methylation occurs at CG or CNG sites, where the complementary base pairing of cytosine and guanine provides the sequence context for “symmetric” methylation on both strands. Non-CpG methylation at CNN sites is called “asymmetric” methylation [18].

Different types of DNA methyltransferases (DNMTs), with different specificities and properties of their activity modulations, catalyze changes in the methylation of CG, CNG, and CNN sites [15]. Changes in the methylation status of CG sites are allele-specific, which can play an important role in organizing the formation of the transcriptional complex and gene activity. CNN sites are targets in the implementation of RNA-dependent DNA methylation (RdDM) [16]. A study on *Arabidopsis* showed the overall methylation status of genomic DNA in the CG, CNG, and CNN sites [15,18]. The alteration of site-specific cytosine methylation status has important implications in plants, including the coordination of gene expression. The dynamics of the locus-specific promoter methylation of membrane subunits of SDH during maize germination may play an important role in the specific mechanism of transcriptional activity control via RdDM. The distribution of cytosine by sites is essential in this process, and for plant organisms, the main target for methylation can be CNG sites [19,20]. The maintenance of DNA methylation takes place in a sequence-dependent manner: the DNMT1 ortholog MET1 maintains the methylation of CG sites. At CNG sites, methylation is carried out with the participation of chromomethylases CMT2 and CMT3. At CNN sequences, DNA methylation is maintained by constant de novo RdDM (de novo methylation refers to methylation of previously unmethylated DNA), catalyzed by DOMAINS REARRANGED METHYLTRANSFERASE 2 (DRM2), or by RdDM-independent methylation, carried out by CMT2 [20,21]. In maize, this type of methylation is performed by ZMET2 [22], which is similar to *Arabidopsis* chromomethylases CMT1 and CMT3, and is involved in maintaining CNG and CNN methylation patterns.

Seed germination is associated with the transition of the organism to the autotrophic type of metabolism, which requires the significant restructuring of not only metabolic processes, including those at the enzyme level, but also their coordination at the genetic and epigenetic levels. In the scutellum, as an oil-storing organ of cereal seeds, the content of triacylglycerols can reach up to 30% of the scutellum mass. The oxidation of fatty acids through glyoxysomes yields, via isocitrate lyase reaction, high amounts of succinate, which is converted by SDH in mitochondria [23,24]. The polymorphism of the SDH subunit composition [25] plays an important role in energy metabolism and its regulation, especially at the level of the mitochondrial ETC [26]. The anchoring of SDH in the mitochondrial membrane represents an important regulatory mechanism of its operation, both as the TCA cycle enzyme and as part of the mitochondrial ETC. No previous studies have explored the locus-specific methylation dynamics of SDH membrane subunit promoters during maize germination. In this regard, we performed a study of the changes in the degree of cytosine methylation in promoters of the genes encoding the membrane-bound SDH subunits during the germination of maize seeds. We demonstrated that the changes in cytosine methylation at the specific sites play a role in the regulation of the expression of *Sdh* genes in the course of the conversion of succinate formed in the glyoxylate cycle.

## 2. Results

### 2.1. Analysis of the Cytosine Methylation Status in Symmetric and Asymmetric Sites of the Promoter of the Sdh3-1 and Sdh3-2 Genes

The evaluation of nucleotide sequences of *Sdh3-1* gene promoter fragments after bisulfite sequencing allowed us to identify the degree of its methylation on days 1, 4, and 8 of seed germination. A higher level of methylation of the CG sites on the first day of germination underwent a marked decrease on the 8th day. A similar trend of change in the cytosine methylation status of CG sites was also established for the *Sdh3-2* gene, with its generally higher degree of methylation (Table 1; Supplementary Figures S1 and S2).

**Figure 1:**
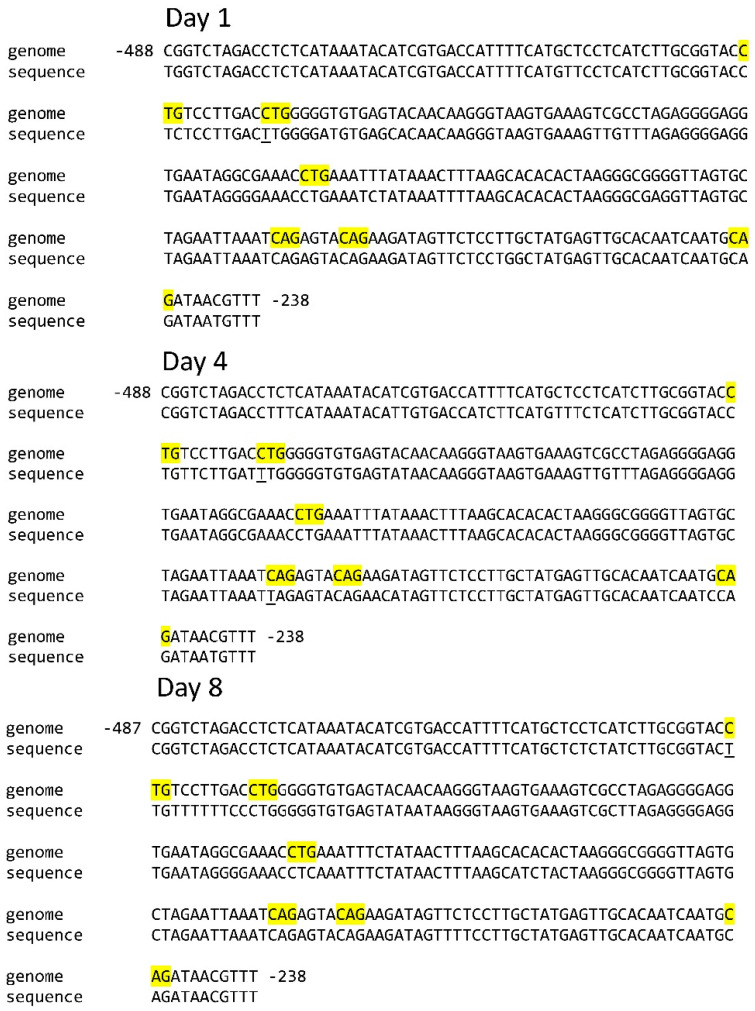
Distribution of CNG sites in the *Sdh3-1* gene promoter and cytosine methylation status in scutella during germination of maize seeds. Thymine, formed from cytosine during bisulfite conversion of DNA, is underlined. “Genome” is the genomic DNA sequence of the promoter of the gene under study, taken from the NCBI database. “Sequence” is the sequence of the amplicon obtained using primers for bisulfite sequencing to the corresponding gene promoter. The highlighted regions on the sequence are the analyzed cytosine methylation sites.

Of the six CNG sites in the analyzed sequence of the *Sdh3-1* gene promoter, only one site was unmethylated on the first day of germination (Table 1). A similar status of CNG sites was shown on the 8th day, but in this case, cytosine in a different position was found in the unmethylated state (Figure 1). On the 4th day, two CNG sites were unmethylated.

For the *Sdh3-2* gene promoter, twelve CNG sites have been identified. On the first day of seed germination, three CNG sites were unmethylated. As maize seeds germinated, two unmethylated CNG sites were found in the scutella on day 4. On the 8th day of seed germination, eight unmethylated CNG sites were found. Consequently, the change in the cytosine methylation status of the *Sdh3-1* and *Sdh3-2* gene promoters in the scutella during maize seed germination is more distinctive for the *Sdh3-2* gene.

**Figure 2:**
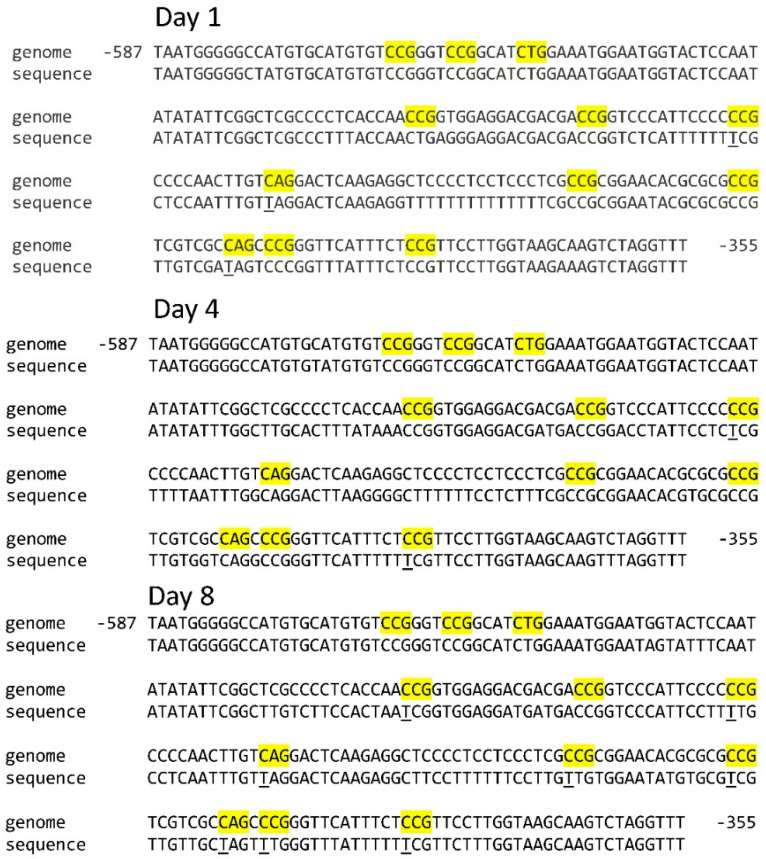
Distribution of CNG sites in the *Sdh3-2* gene promoter and cytosine methylation in scutella during germination of maize seeds. Thymine, formed from cytosine during bisulfite conversion of DNA, is underlined. “Genome” is the genomic DNA sequence of the promoter of the gene under study, taken from the NCBI database. “Sequence” is the sequence of amplicon obtained using primers for bisulfite sequencing to the corresponding gene promoter. The highlighted regions on the sequence are the analyzed cytosine methylation sites.

The analysis of the position of methylated and unmethylated CNG sites of the *Sdh3-1* gene promoter showed that there were minor changes in the redistribution of the cytosine methylation status. The sites at positions −249, −289, and −353 were constantly methylated, while the methylation status of the three CNG sites at positions −296, −417, and −428 changed. The site at position −296 was methylated on days 1 and 8, the site at position −417 was only methylated on day 8, and the site at position −428 was methylated on days 1 and 4 (Figure 1). A different pattern of the redistribution of methylated and unmethylated CNG sites occurs for the *Sdh3-2* gene promoter of the SDH subunit C in the course of germination. The CNG sites at positions −485, −550, −557, and −563 remained methylated throughout the entire studied period (Figure 2).

The analysis of the nucleotide sequence of the *Sdh3-1* gene promoter sequence on different days of germination showed a change in the cytosine methylation status in several CNN sites (Table 1). On the first day, 6 out of 36 CNN sites were unmethylated at cytosine, which was detected based on the sequence after the bisulfite conversion of DNA. A study of the status of asymmetric sites revealed that as seeds developed, there was a gradual but small decrease in the methylation level of the CNN sites in the *Sdh3-1* gene promoter, and by the 8th day of germination, the methylation level decreased to ~75%, compared to the level of 83% seen on the first day.

The number of CNN sites in the *Sdh3-2* gene promoter was found to be 48, with 26 of them (~46%) being unmethylated during the studied period of germination. Major changes in the methylation status of the CNN sites of the *Sdh3-2* gene promoter were observed during germination (46% on the first day, 42% on the 4th day, and 56% on the 8th day) (Table 1).

**Figure 3:**
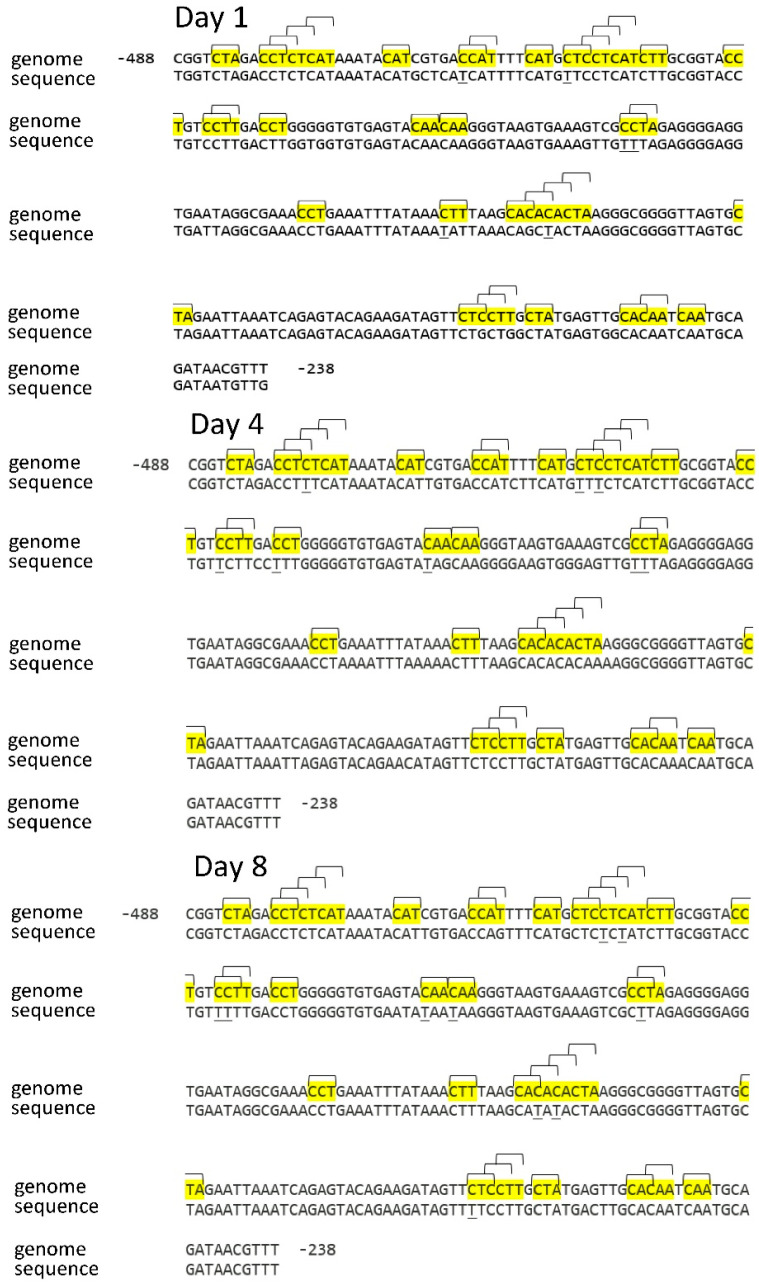
Distribution of CNN sites in the *Sdh3-1* gene promoter and cytosine methylation status in scutella during germination of maize seeds. Thymine, formed from cytosine during bisulfite conversion of DNA, is underlined. “Genome” is the genomic DNA sequence of the promoter of the gene under study, taken from the NCBI database. “Sequence” is the sequence of the amplicon obtained using primers for bisulfite sequencing to the corresponding gene promoter. The highlighted regions on the sequence are the analyzed cytosine methylation sites. Brackets indicate methylation sites and their positions on the sequence.

**Figure 4:**
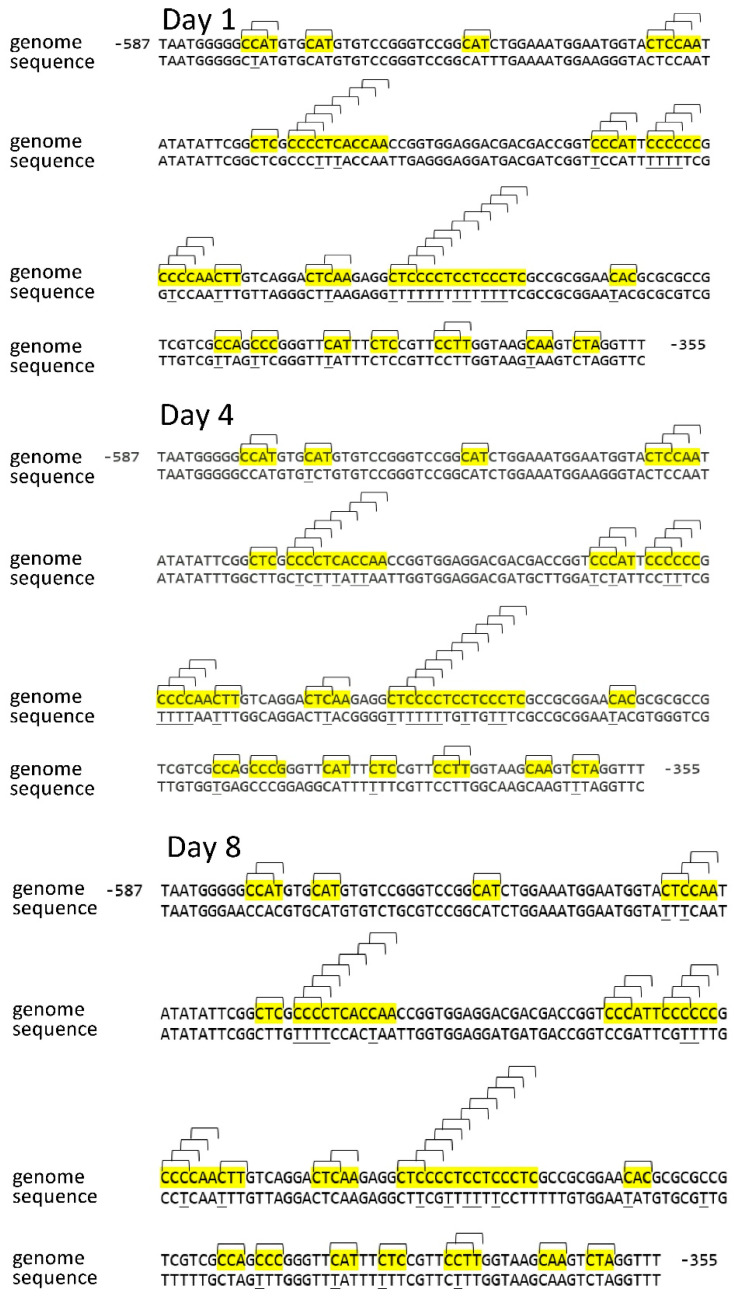
Distribution of CNN sites in the *Sdh3-2* gene promoter and cytosine methylation status in scutella during germination of maize seeds. Thymine, formed from cytosine during bisulfite conversion of DNA, is underlined. “Genome” is the genomic DNA sequence of the promoter of the gene under study, taken from the NCBI database. “Sequence” is the sequence of the amplicon obtained using primers for bisulfite sequencing to the corresponding gene promoter. The highlighted regions on the sequence are the analyzed cytosine methylation sites. Brackets indicate methylation sites and their positions on the sequence.

The change in the cytosine methylation status in the CNN site in the *Sdh3-1* and *Sdh3-2* genes thus reveals an opposite pattern. The *Sdh3-1* gene is characterized by a slight decrease in the studied indicator, while the *Sdh3-2* gene shows an increase in the percentage of methylation of the promoter on the 8th day of germination (Figure 3 and Figure 4).

### 2.2. Analysis of the Cytosine Methylation Status in Symmetric and Asymmetric Sites of the Promoters of the SDH Subunit D Gene

**Figure 5:**
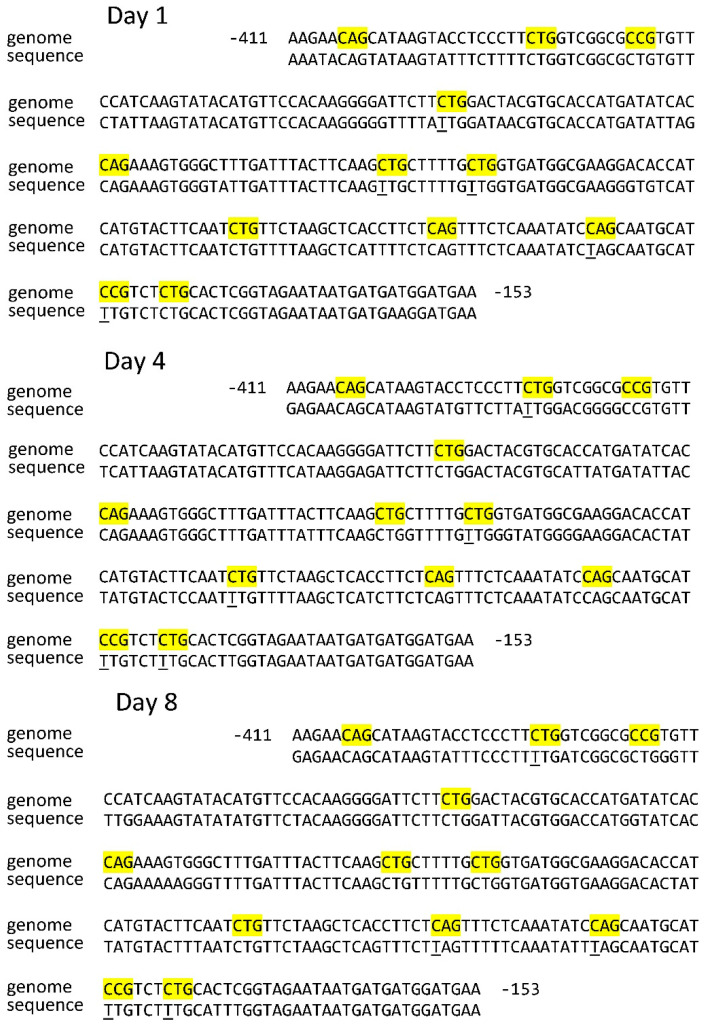
The distribution of CNG sites in the *Sdh4* gene promoter and the cytosine methylation status in scutella during the germination of maize seeds. Thymine, formed from cytosine during bisulfite conversion of DNA, is underlined. “Genome” is the genomic DNA sequence of the promoter of the gene under study, taken from the NCBI database. “Sequence” is the sequence of the amplicon obtained using primers for bisulfite sequencing to the corresponding gene promoter. The highlighted regions on the sequence are the analyzed cytosine methylation sites.

**Figure 6:**
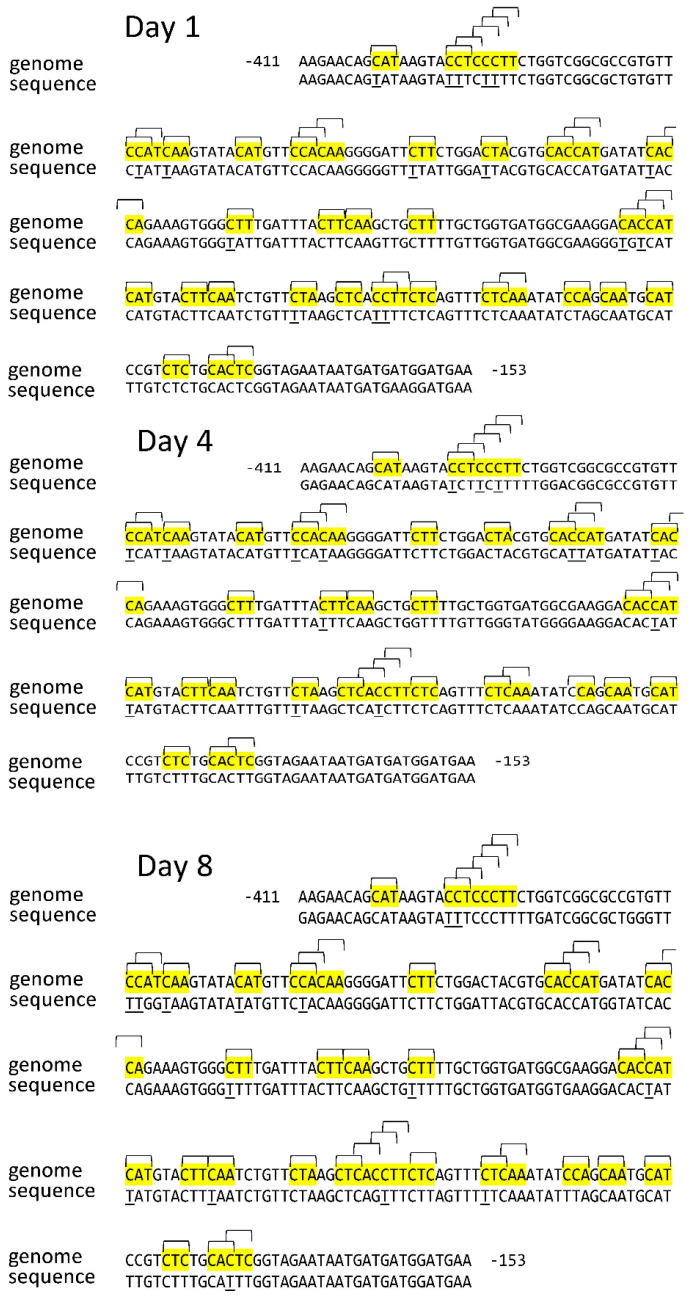
The distribution of CNN sites in the *Sdh4* gene promoter and the cytosine methylation status in scutella during the germination of maize seeds. Thymine, formed from cytosine during bisulfite conversion of DNA, is underlined. “Genome” is the genomic DNA sequence of the promoter of the gene under study, taken from the NCBI database. “Sequence” is the sequence of the amplicon obtained using primers for bisulfite sequencing to the corresponding gene promoter. The highlighted regions on the sequence are the analyzed cytosine methylation sites. Brackets indicate methylation sites and their positions on the sequence.

The cytosine methylation status in the asymmetric methylation sites of the *Sdh4* gene promoter revealed only minor changes throughout the entire period of seed germination. This included an increase from 2 to 4 in the number of unmethylated CG sites on day 8, and a decrease from 16 to 15 in unmethylated CNN sites on days 4 and 8 as compared to day 1 (Table 1, Figure 5 and Figure 6, Supplementary Figure S3).

However, the distribution pattern of methylated cytosines in the promoter sequence of the *Sdh4* gene revealed more significant changes. The CNN sites at positions −184, −366, and −395 were unmethylated throughout the entire period of seed germination. The asymmetric sites that were constantly in a methylated state in the *Sdh4* gene promoter were uniformly distributed along the promoter sequence. A significant portion of the CNN sites changed methylation status at different stages of maize seed germination. Six sites (−237, −221, −237, −349, −352, and −392) changed their methylation status in a wave-like manner, being methylated on days 1 and 8 of seed germination. Three sites (−201, −222, and −299) changed their methylation status in opposite directions, being methylated on day 4.

Between the first and fourth days of seed germination, no significant changes in the redistribution of the cytosine methylation status between the analyzed sites were observed (Figure 5). On the 8th day, an almost twofold decrease in the methylation level of CG sites was observed, with a concomitant increase in the percentage of the methylation of CNN sites, indicating the primary role of CNN sites during this period in the control of the DNA methylation status by RdDM [15]. The distribution of methylcytosine between the CNG and CNN sites within the promoter of the SDH subunit D gene only slightly changed during germination (Figure 5 and Figure 6).

### 2.3. Changes in the Methylation Patterns of Sdh3-2, Sdh3-2, and Sdh4 Genes During Germination

**Figure 7:**
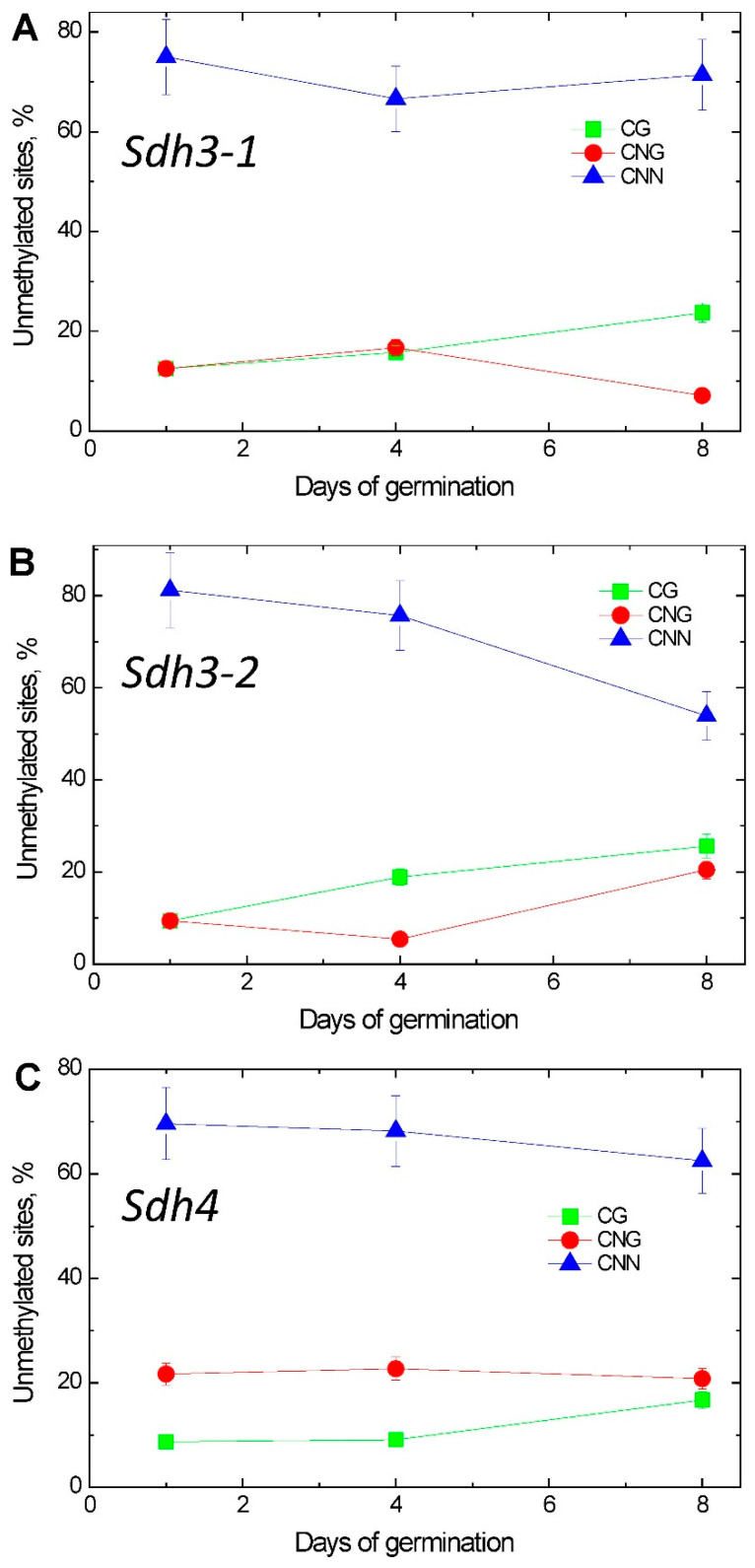
Changes in the methylation patterns of (**A**) *Sdh3-1*, (**B**) *Sdh3-2*, and (**C**) *Sdh4* genes during germination.

The calculation of the numerical values of the methylation status of the promoters of the studied genes was performed based on a comparison of the nucleotide sequences of the promoter before and after bisulfite conversion. The values of the degree of promoter methylation were estimated by C-to-T substitutions of specific cytosines in the analyzed sites [27], and the % of the total amount of symmetric and asymmetric sites was calculated. In the first days of seed germination, the proportion of unmethylated cytosines among all methylation sites was distributed as follows: 12.5% in CG, 12.5% in CNG, and 75.0% in CNN. Consequently, the main methylation occurs at CNG sites, and the smallest contribution to the total cytosine methylation during this period of seed germination is made by cytosines in CNN (Figure 7). On the 4th day of maize seed germination, the distribution of the proportion of unmethylated cytosines by symmetric and asymmetric sites only changed slightly. Then, the proportion of cytosine methylation at CG dinucleotide sites increased, but the proportion of methylation of CNG sites decreased by a similar amount, which indicates that during this period of seed germination, a redistribution of methylation status occurs between two types of methylation sites—CG and CNG.

The cytosine methylation status of the *Sdh3-2* promoter undergoes major changes during seed germination in the context of various methylation sites: CG, CNG, and CNN. On the first day of seed germination, a significant proportion of unmethylated cytosines was seen in CNN sites (81%); the rest (9.5% each) accounted for cytosines in CG and CNG sites. On the 4th day, a significant redistribution of the cytosine methylation status between CG and CNG sites occurred. On the 8th day, the distribution of the proportion of unmethylated cytosines in the *Sdh3-2* promoter indicated the significant redistribution of the cytosine methylation status within the analyzed sites. A significant decrease in the unmethylated CNN sites was observed, with a corresponding increase in the proportion of unmethylated cytosines in CG and CNG sites (Figure 7B). The changes in cytosine methylation status of the *Sdh3-1* promoter are much less pronounced (Figure 7A), and the changes in cytosine methylation status of the *Sdh4* are negligible (Figure 7C).

### 2.4. Changes in the Activity of Cytosine DNA Methyltransferases During Germination

**Figure 8:**
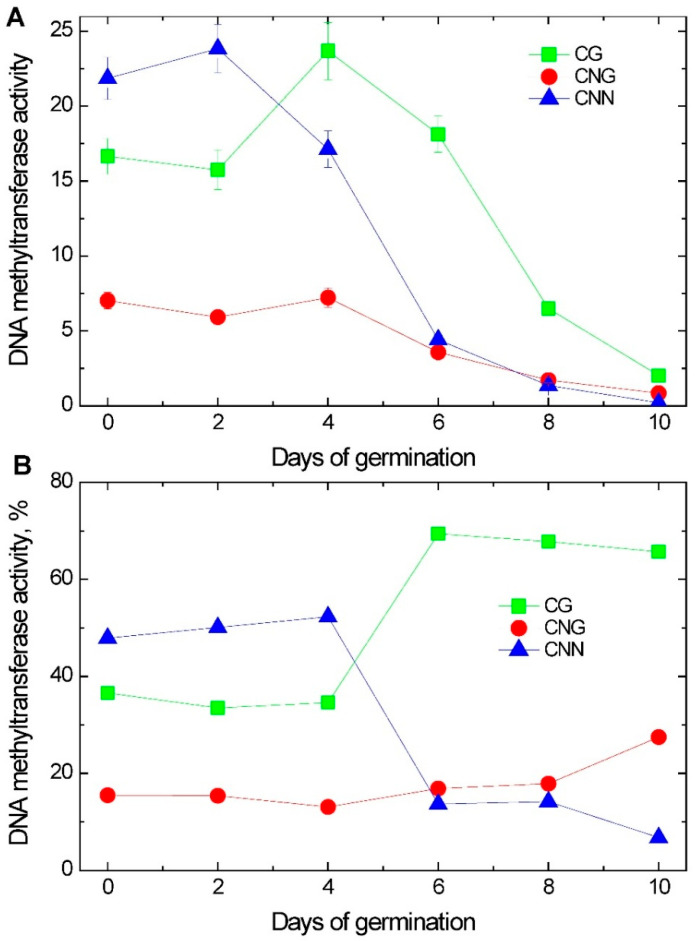
Changes in CG-, CNG-, and CNN-specific DNA cytosine methyltransferase activities during germination: (**A**) total activity in μmol *S*-adenosylhomocysteine per minute; (**B**) % activity of each DNA methyltransferase.

We studied the total activity of the corresponding site-dependent DNA methyltransferases. It is known that MET1 maintains the methylation of CG sites; at CNG, sites methylation is maintained by CMT2 and CMT3, and at CNN sequences, it is carried out by RdDM-dependent methylation with the participation of DRM2, CMT2, and ZMET2 in maize. Figure 8A shows the change in the activity of the corresponding DNA methyltransferases during the germination of maize seeds, while Figure 8B shows the contribution of each type of site-dependent DNA methyltransferase under the same experimental conditions. The change in the activity of DNA methyltransferases during germination indicates the intensity of catalysis of each enzyme at different stages of germination, while Figure 8B shows the ratio of their activities, which indicates a change in the type of methylation at the initial and late stages of the germination of maize seeds.

The study of the DNA methyltransferases methylating CG sites revealed that their high level of activity was observed at the beginning of germination, with the maximum on the 4th day, after which the activity decreased. The CNG DNA methyltransferases exhibit a similar profile with a lower activity and its less pronounced changes (Figure 8A). The activity of DNA methyltransferases methylating CNN sites has the maximum value on the 2nd day of germination, decreasing by the 10th day of germination by more than a hundred times. This which the most pronounced change among the DNA methyltransferases (Figure 8). Until the 4th day of seed germination, there were no significant changes in the redistribution of cytosine DNA methyltransferase activities. Then, the contribution of CNN DNA methyltransferases decreased, and the input of CNG DNA methyltransferases to the total activity increased almost twice (Figure 8B).

### 2.5. Changes in Isocitrate Lyase Activity and Expression

**Figure 9:**
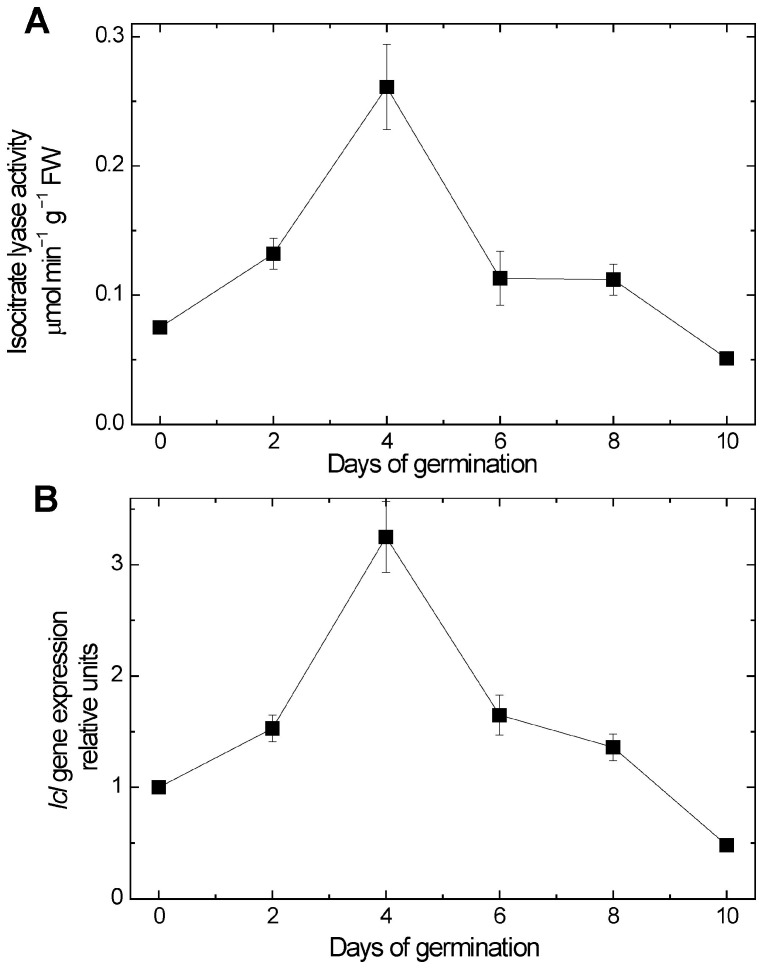
Changes in isocitrate lyase activity (**A**) and in expression of *Icl* gene (**B**) during germination.

A study of the dynamics of isocitrate lyase activity in maize scutella showed that its induction is observed from the first days of germination, reaching its maximum value on the 4th day (Figure 9A). The activity pattern represents a characteristic bell-shaped curve. It correlated well with the values of expression of the *Icl* gene, encoding isocitrate lyase (Figure 9B).

## 3. Discussion

Changes in the expression of *Sdh* genes, including hydrophobic subunits that are important for anchoring the enzyme in the inner mitochondrial membrane, are associated with various mechanisms. One of these mechanisms is epigenetic regulation, caused by the changes in the methylation status of the regulatory regions of the *Sdh3-1* and *Sdh3-2* genes, which encode the C subunit of SDH. Previously, the type of mechanism for controlling the expression of *Sdh* genes was identified for *Sdh1-1* and *Sdh1-2* of the SDH flavoprotein during maize seed germination [7,25]. Our earlier studies of the level of transcripts of the *Sdh* genes indicate that their differential expression during germination results in corresponding changes in SDH activity [7,8]. The current study allows us to conclude that the change in the methylation of the *Sdh* gene promoters in the scutella during the germination of maize seeds is associated with the redistribution of the methyl status of the cytosine between symmetrical (CG and CNG) sites and within the asymmetric CNN site. The observed increase in the cytosine methylation level of the *Sdh* gene promoters can contribute to the regulation of their expression.

The fine control of *Sdh* gene expression is particularly important during the germination of oil-storing seeds, when the β-oxidation of fatty acids and further conversion of acetyl-CoA in the glyoxylate cycle result in the formation of large quantities of succinate. At that time, succinate is formed in both the tricarboxylic acid cycle and in the glyoxylate cycle, and the flux through the glyoxylate cycle at the peak of its activity significantly exceeds the flux through the TCA cycle [23,24]. Isocitrate lyase provides the operation of the glyoxylate cycle, which is a stage of gluconeogenesis, and forms succinate, i.e., it ensures the mobilization of reserve lipids in germinating seeds. The induction of isocitrate lyase thus can indicate the intensification of biosynthetic metabolic processes, including gluconeogenesis, in oil-storing tissues during seed germination, for the operation of which the coordination of the *Sdh* genes can exert a control over the transcriptional activity of genes and the synthesis of the corresponding SDH isoenzymes [24,25]. Both an increase in activity and an increase in the expression of the *Icl* gene were observed during germination, with the peak on day 4, following the decline (Figure 9A,B). Although there is an indication that the excess of succinate can be converted in glyoxysomes by an oxidase having low affinity to succinate [11], the main route of succinate conversion is its oxidation in mitochondria by SDH. The synthesis of the SDH subunits at this time is necessary for the effective operation of mitochondrial metabolism and the intensification of the mobilization of succinate formed in the glyoxylate cycle.

Cytosine methylation is a unique regulatory feature of both plant and animal genomes. A large number of seed genes are regulated via methylation changes during development, likely due to the action of specific transcription factors and epigenetic events at the chromatin level [28]. It has been previously demonstrated that in some cases, genome-wide methylation of the CG and CNG sites may not change significantly during seed development [29,30]. Nevertheless, seed germination results in major changes in DNA methylation level, which is of great importance for seedling development [31]. Changes in DNA methylation have been shown for several plants during their development, which is associated with the coordination of metabolism during the transition to photosynthetic activity [32].

The conducted analysis of the change in the activity of cytosine DNA methyltransferases in the scutella of maize seeds during their germination allowed us to identify the relative contribution of CG, CNG, and CNN DNA methyltransferases (Figure 8B). At the initial stages of maize seed development, the predominant activity of CNN DNA methyltransferases is observed, accounting for more than 85% of the total cytosine methylating activity. This is largely associated with the type of control of the DNA methylation status by the mechanism of RdDM [19]. However, as the seeds develop, there is a redistribution of the activity values between CG and CNN DNA methyltransferases towards an increase in the proportion of CG DNA methyltransferases. During this period of seed germination, the main contribution to the total DNA methylation in maize scutella is made by CG DNA methyltransferase sites, largely associated with the type of control of the DNA methylation by CpG islands [33]. This type of methylation of sites is important in the reorganization of transcriptional activity in the regions of CpG islands. The status of CG dinucleotides plays an important role in interaction with transcription factors, including through participation in chromatin reorganization [34]. Cytosines located in the TSS region significantly affect interaction with transcription factors, which is due to their methylation status [35,36,37].

**Figure 10:**
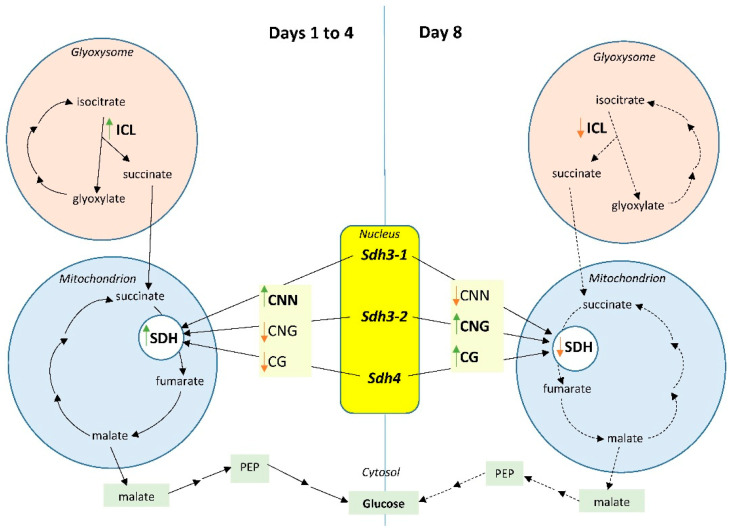
Scheme of epigenetic regulation of succinate dehydrogenase caused by changes in the cytosine methylation status of symmetric and asymmetric sites of the *Sdh3-1*, *Sdh3-2*, and *Sdh4* gene promoters in maize scutella during seed germination. Abbreviations: SDH, succinate dehydrogenase; ICL, isocitrate lyase; CG and CNG, symmetric cytosine methylation sites; CNN, asymmetric cytosine methylation sites. Solid lines indicate active metabolic processes; dotted lines indicate less active metabolic processes. Green arrows show activation of the enzymes (ICL, SDH, and methyltransferases) and the increase in site methylation. Red arrows show the inactivation of the corresponding enzymes as well as the decrease in site methylation.

The dynamics of changes in the activity of cytosine DNA methyltransferases at symmetric and asymmetric sites in DNA during germination of maize seeds indicate the important role of this epigenetic mechanism in the regulation of the transcriptional activity of the genome. The study of the dynamics of the cytosine methylation status in symmetric and asymmetric sites of the *Sdh* gene promoters revealed its ontogenetic changes, suggesting that SDH plays an essential role in the metabolism of developing seeds when the type of nutrition changes from heterotrophic to autotrophic (Figure 10). At the initial stages of germination, the mobilization of reserve substances occurs in the glyoxysome via the glyoxylate cycle. Gluconeogenic processes in the cytosol ensure the mobilization of reserve lipids into an accessible form of carbohydrates with the participation of SDH in mitochondria. At the same time, CNN sites play a major role in regulating the transcriptional activity of the *Sdh* genes, the methyl status of which is regulated by the CNN DNA methyltransferase. As the plant organism develops, its type of nutrition changes to autotrophic, and the decrease in the intensity of transcription of the *Sdh* genes indicates a switch in the energy metabolism of the plant cell to photosynthetic activity. The obtained data show that on the 8th day of seed germination, the activity of CG and CNG DNA methyltransferases predominates, which correlates with an increase in the proportion of CG and CNG sites in the total cytosine methylation status of the promoters of the studied genes.

The conducted studies allow us to conclude that the change in methylation of the *Sdh* gene promoters in the scutella during the germination of maize seeds is associated with the redistribution of the cytosine methylation status between symmetric (CG and CNG) and asymmetric sites (CNN). At the same time, in the symmetric CNG sites, the change in the cytosine methylation status throughout the entire period of seed germination was insignificant. Consequently, the main contribution to the epigenetic mechanism of regulation of the transcriptional activity of the *Sdh* genes in the scutella of germinating maize seeds is made by cytosine CG sites, and to a lesser extent, CNG, the methylation of which is carried out by the enzymes MET1 and CMT3, respectively [15]. The change in the cytosine methylation status in the CNG and CNN sites is less pronounced in the overall methylation status of the promoters of the studied genes, compared to CG methylation. Since CNG and CNN sites are methylated via RdDM, mediated by the DNA methyltransferase CMT2 [20,21], this type of epigenetic control is weakly exerted during maize seed germination, especially at later stages.

## 4. Materials and Methods

### 4.1. Object of Investigation

The object of the study was the scutella of maize (*Zea mays* L., cv Voronezhskaya 76) plants grown hydroponically at 25 °C for 8 days with 12 h daylight of intensity 90 μmol quanta m^−2^ s^−1^ at 25 °C.

### 4.2. DNA Extraction and Bisulfite Sequencing

DNA extraction was performed by the phenol-chloroform method [38]. The quality of nucleic acids was confirmed electrophoretically in a 1% agarose gel.

The MethPrimer program (http://www.urogene.org/methprimer/index1.html, accessed on 20 October 2023) was used to analyze the *Sdh* gene promoters for the presence of symmetric and asymmetric cytosine methylation sites and to select bisulfite sequencing primers. The nucleotide sequences of the promoter regions of the maize *Sdh* genes were taken from the NCBI database (USA, http://www.ncbi.nlm.nih.gov), and the nucleotide sequence of approximately 1000 nucleotides located upstream of the start codon was taken from the corresponding gene. Primer sequences for bisulfite sequencing: to the promoter of the gene *Sdh3-1* (LOC100281355): forward 5′-TTTGTTATGAGTTGTATAATTAA-3′, reverse 5′-TTATAAAACTTACAAAAAACACCTAATTAT-3′; to the promoter of the gene *Sdh3-2* (LOC100283351): forward 5′-TAGATAATTTGAAAATTTTTGGATA-3′; reverse 5′-AATATCAATACCCAAACAACAAAATC-3′; to the promoter of the gene *Sdh4* (LOC100280324): forward 5′-TTTTAAAGTTTTTATTTTTTTTGA-3′; reverse 5′-GTATTAGGCGGTTTTAGAGAAGG-3′.

To analyze the cytosine methylation status, bisulfite conversion of DNA [39] was performed, followed by bisulfite sequencing. Polymerase chain reaction with primers for bisulfite sequencing was performed using the Thermo Scientific DremTaq PCR MasterMix (2×) reagent kit (Thermo Fisher Scientific, Waltham, MA, USA). The PCR reaction was carried out on a Tertsik multichannel thermal cycler (DNA-Technology, Moscow, Russia) with the following amplification parameters: preliminary denaturation at 95 °C for 10 min, then 35 cycles: 95 °C—20 s, 52 °C—20 s, 72 °C—30 s, and finally 72 °C—4 min.

The methylation status and the distribution pattern of methylated cytosine in CG, CNG, and CNN sites of the *Sdh3-1*, *Sdh3-2*, and *Sdh4* genes were assessed on days 1, 4, and 8 of germination. The primers were selected taking into account the assessment of the region located immediately above the transcription initiation zone (transcription start site, TSS) [17]. For the *Sdh3-1* gene promoter, the promoter region for analysis corresponded to the position −238…−488, for *Sdh3-2*, −355…−587, and for *Sdh4*, −153…−411.

The amplification products were sequenced at Evrogen (Moscow, Russia). The numerical values of the methylation status of the promoter of the gene under study were calculated based on a comparison of the nucleotide sequences of the promoter before and after bisulfite conversion. The values of the degree of promoter methylation were estimated by C-to-T substitutions [27] and calculated from the total sum of symmetric and asymmetric cytosine methylation sites.

### 4.3. DNA Methyltransferase Activity

The activity of cytosine DNA methyltransferases was determined spectrophotometrically in a medium containing: 10 mM Tris-HCl, pH 7.9, 50 mM NaCl, 10 mM MgCl_2_, 1 mM DTT, 80 μM *S*-adenosylhomocysteine (SAM), 40 nM DNA [40]. The following nucleotide sequences were used as substrates for different types of DNA methyltransferases:

For methylation analysis of CG-sites: CGACGACGTTAAAATACGAAAT

For methylation analysis of CNG-sites: AAACCGAACCGAAAAACCG

For methylation analysis of CNN-sites: TACAACCAAAAAAACCTCTTC

The enzyme activity was assessed by the change in the optical density of the photometric medium at a wavelength of 256 nm for the formation of *S*-adenosylhomocysteine during the reaction [41]. The amount of enzyme catalyzing the formation of 1 μmol of *S*-adenosylhomocysteine per minute during the reaction was taken as a unit of enzymatic activity.

### 4.4. Isocitrate Lyase Activity and Expression

The activity of isocitrate lyase was determined spectrophotometrically on SF-2000 (OKB Spectr, Moscow, Russia) by the change in light absorption at 324 nm due to the formation of a complex of phenylhydrazine with glyoxylate [42]. The spectrophotometric medium contained 50 mM Tris-HCl buffer, pH 7.5, 5 mM MgCl_2_, 4 mM dithiothreitol, 2 mM sodium isocitrate, and 4 mM phenylhydrazine hydrochloride. The activity was measured at 25 °C.

To determine changes in the expression level of the maize *Icl* gene (LOC103633249), qRT-PCR was performed on a LightCycler 96 device (Roche, Solna, Sweden) using SYBR Green I as a dye. Primers were selected based on a comparison of the nucleotide sequences of the isocitrate lyase gene using Primer-Blast software—https://www.ncbi.nlm.nih.gov/tools/primer-blast/, accessed on 20 October 2023. The primers for qRT-PCR analysis were as follows: forward—5′-CTACGACAGGGTGCTCAAGG-3′; reverse—5′-TTTGGCCATAACTTGCAGGC-3′. Amplification parameters were as follows: preliminary denaturation at 95 °C for 5 min. There were 35 cycles, 95 °C—20 s, 58 °C—20 s, and 72 °C—15 s (detection), and final elongation at 72 °C—4 min.

### 4.5. Statistical Data Processing

The experiments were carried out in three biological and four analytical replicates. The data were subjected to two-way analysis of variance (ANOVA) using STATISTICA data analysis software version 9.0 (Statsoft Wipro, East Brunswick, NJ, USA). The results are presented as mean values and standard deviations (SD). Statistically significant differences are discussed at *p* < 0.05 [43].

## 5. Conclusions

Changes in the cytosine methylation status of the promoters of the genes encoding the membrane-bound SDH subunits in the scutella of germinating maize seeds contribute to the regulation of the expression of *Sdh* genes in the course of the conversion of succinate formed in the glyoxylate cycle. The obtained data reveal that the changes in methylation of the *Sdh* gene promoters in the scutella of maize seeds during germination are associated with the redistribution of the cytosine methylation between symmetric (CG and CNG) and asymmetric (CNN) sites. The main contribution is made by the cytosine of the CG sites, the methylation of which is controlled by MET1 enzymes. The change in the methyl status of the CG sites is allele-specific, which can play a role in organizing the formation of the transcriptional complex and gene activity. CNN sites are the targets for RNA-dependent DNA methylation. The difference in the mechanism of the methylation regulation of different cytosine sites provides the multidirectional control of the transcriptional activity of *Sdh* genes. During seed development, there is a change in nutrition types from heterotrophic to autotrophic; as such, the *Sdh* gene system controlled by an epigenetic mechanism can play a special role in adapting to metabolic changes.

## Figures and Tables

**Table 1:** Quantitative parameters of cytosine methylation in symmetric and asymmetric sites of promoters of *Sdh* genes in maize scutella during seed germination.

	*Sdh3-1*			*Sdh3-2*			*Sdh4*		
	Day 1	Day 4	Day 8	Day 1	Day 4	Day 8	Day 1	Day 4	Day 8
CG sites									
Number of CG	7	7	7	20	20	20	7	7	7
Number of un-methylated CG	4	4	6	2	5	11	2	2	4
Methylation (%)	42.9	42.9	14.3	90.0	75.0	45.0	71.4	71.4	42.9
CNG sites									
Number of CNG	6	6	6	12	12	12	12	12	12
Number of un-methylated CNG	1	2	1	3	2	8	5	5	5
Methylation (%)	83.3	66.7	83.3	75.5	83.3	33.3	58.3	58.3	58.3
CNN sites									
Number of CNN	36	36	36	48	48	48	43	43	43
Number of un-methylated CNN	6	8	10	26	28	21	16	15	15
Methylation (%)	83.3	77.7	75.6	45.8	41.7	56.3	62.8	65.1	65.1
Bisulfite conver-sion of DNA (%)	87.8	84.1	85.4	81.1	87.7	84.5	78.3	80.7	81.0

## Data Availability

The datasets generated for this study are available upon request from the corresponding author.

## Supplementary Materials

The following supporting information can be downloaded at: https://www.mdpi.com/article/10.3390/ijms26168010/s1.

## Abbreviations

CMT	chromomethylase
DNMT	DNA methyltransferase
DRM	DOMAINS REARRANGED METHYLTRANSFERASE
RdDM	RNA-dependent DNA methylation
SDH	succinate dehydrogenase
TCA cycle	tricarboxylic acid cycle
