# Supplementary material for: Site-Specific Changes in Cytosine Methylation in Promoters of the Genes Encoding the Membrane Subunits of Succinate Dehydrogenase During Germination of Maize Seeds
Source: Int J Mol Sci. 2025 Aug 19;26(16):8010. doi: 10.3390/ijms26168010 (PMC12386843; doi:10.3390/ijms26168010)
Supplement: Supplementary file 1 [file ijms-26-08010-s001.zip › ijms-3756572-supplementary.pdf]

Sdh3-1

Day 1

```
genome     -488 CGGTCTAGACCTCTCATAAATACATCGTGACCATTTTCATGCTCCTCATCTTGCGGTACC
sequence        TGGTCTAGACCTCTCATAAATACATCGTGACCATTTTCATGTTCCTCATCTTGCGGTACC

genome          TGTCCTTGACCTGGGGGTGTGAGTACAACAAGGGTAAGTGAAAGTCGCCTAGAGGGGAGG
sequence        TCTCCTTGACTTGGGGATGTGAGCACAACAAGGGTAAGTGAAAGTTGTTTAGAGGGGAGG

genome          TGAATAGGCGAAACCTGAAATTTATAAACTTTAAGCACACACTAAGGGCGGGGTTAGTGC
sequence        TGAATAGGCGAAACCTGAAATCTATAAATTTTAAGCACACACTAAGGGCGAGGTTAGTGC

genome          TAGAATTAAATCAGAGTACAGAAGATAGTTCTCCTTGCTATGAGTTGCACAATCAATGCA
sequence        TAGAATTAAATCAGAGTACAGAAGATAGTTCTCCTGGCTATGAGTTGCACAATCAATGCA

genome          GATAACGTTT -238
sequence        GATAATGTTT
```

Day 4

```
genome     -488 CGGTCTAGACCTCTCATAAATACATCGTGACCATTTTCATGCTCCTCATCTTGCGGTACC
sequence        CGGTCTAGACCTTTCATAAATACATTGTGACCATCTTCATGTTTCTCATCTTGCGGTACC

genome          TGTCCTTGACCTGGGGGTGTGAGTACAACAAGGGTAAGTGAAAGTCGCCTAGAGGGGAGG
sequence        TGTTCTTGATTTGGGGGTGTGAGTATAACAAGGGTAAGTGAAAGTTGTTTAGAGGGGAGG

genome          TGAATAGGCGAAACCTGAAATTTATAAACTTTAAGCACACACTAAGGGCGGGGTTAGTGC
sequence        TGAATAGGCGAAACCTGAAATTTATAAACTTTAAGCACACACTAAGGGCGGGGTTAGTGC

genome          TAGAATTAAATCAGAGTACAGAAGATAGTTCTCCTTGCTATGAGTTGCACAATCAATGCA
sequence        TAGAATTAAATTAGAGTACAGAACATAGTTCTCCTTGCTATGAGTTGCACAATCAATCCA

genome          GATAACGTTT -238
sequence        GATAATGTTT
```

Day 8

```
genome    -487 CGGTCTAGACCTCTCATAAATACATCGTGACCATTTTCATGCTCCTCATCTTGCGGTACC
sequence       CGGTCTAGACCTCTCATAAATACATCGTGACCATTTTCATGCTCTCTATCTTGCGGTACT

genome         TGTCCTTGACCTGGGGGTGTGAGTACAACAAGGGTAAGTGAAAGTCGCCTAGAGGGGAGG
sequence       TGTTTTTTCCCTGGGGGTGTGAGTATAATAAGGGTAAGTGAAAGTCGCTTAGAGGGGAGG

genome         TGAATAGGCGAAACCTGAAATTTCTATAACTTTAAGCACACACTAAGGGCGGGGTTAGTG
sequence       TGAATAGGTGAAACCTCAAATTTCTATAACTTTAAGCATCTACTAAGGGCGGGGTTAGTG

genome         CTAGAATTAAATCAGAGTACAGAAGATAGTTCTCCTTGCTATGAGTTGCACAATCAATGC
sequence       CTAGAATTAAATCAGAGTACAGAAGATAGTTTTCCTTGCTATGAGTTGCACAATCAATGC

genome         AGATAACGTTT -238
sequence       AGATAACGTTT
```

Supplementary Figure S1. Distribution of CG sites in the *Sdh3-1* gene promoter and the methyl status of cytosine in scutella during germination of maize seeds. Thymine, formed from cytosine during bisulfite conversion of DNA, is underlined. "Genome" is the genomic DNA sequence of the promoter of the gene under study, taken from the NCBI database. "Sequence" is the sequence of the amplicon obtained using primers for bisulfite sequencing to the corresponding gene promoter. The highlighted regions on the sequence are the analyzed cytosine methylation sites.

Sdh3-2

Day 1

```
genome    -587 TAATGGGGGCCATGTGCATGTGTCCGGGTCCGGCATCTGGAAATGGAATGGTACTCCAAT
sequence       TAATGGGGGCTATGTGCATGTGTCCGGGTCCGGCATCTGGAAATGGAATGGTACTCCAAT

genome         ATATATTCGGCTCGCCCCTCACCAACCGGTGGAGGACGACGACCGGTCCCATTCCCCCCG
sequence       ATATATTCGGCTCGCCCTTTACCAACTGAGGGAGGACGACGACCGGTCTCATTTTTTTCG

genome         CCCCAACTTGTCAGGACTCAAGAGGCTCCCCTCCTCCCTCGCCGCGGAACACGCGCGCCG
sequence       CTCCAATTTGTTAGGACTCAAGAGGTTTTTTTTTTTTTCGCCGCGGAATACGCGCGCCG

genome         TCGTCGCCAGCCCGGGTTCATTTCTCCGTTCCTTGGTAAGCAAGTCTAGGTTT    -355
sequence       TTGTCGATAGTCCCGGTTTATTTCTCCGTTCCTTGGTAAGAAAGTCTAGGTTT
```

Day 4

```
genome    -587 TAATGGGGGCCATGTGCATGTGTCCGGGTCCGGCATCTGGAAATGGAATGGTACTCCAAT
sequence       TAATGGGGGCCATGTGTATGTGTCCGGGTCCGGCATCTGGAAATGGAATGGTACTCCAAT

genome         ATATATTCGGCTCGCCCCTCACCAACCGGTGGAGGACGACGACCGGTCCCATTCCCCCCG
sequence       ATATATTTGGCTTGCACTTTATAAACCGGTGGAGGACGATGACCGGACCTATTCCTCTCG

genome         CCCCAACTTGTCAGGACTCAAGAGGCTCCCCTCCTCCCTCGCCGCGGAACACGCGCGCCG
sequence       TTTTAATTTGGCAGGACTTAAGGGGCTTTTTTCCTCTTTCGCCGCGGAACACGTGCGCCG

genome         TCGTCGCCAGCCCGGGTTCATTTCTCCGTTCCTTGGTAAGCAAGTCTAGGTTT    -355
sequence       TTGTGGTCAGGCCGGGTTCATTTTTTCGTTCCTTGGTAAGCAAGTTTAGGTTT
```

Day 8

```
genome    -587 TAATGGGGGCCATGTGCATGTGTCCGGGTCCGGCATCTGGAAATGGAATGGTACTCCAAT
sequence       TAATGGGGGCCATGTGCATGTGTCCGGGTCCGGCATCTGGAAATGGAATAGTATTTCAAT

genome         ATATATTCGGCTCGCCCCTCACCAACCGGTGGAGGACGACGACCGGTCCCATTCCCCCCG
sequence       ATATATTCGGCTTGTCTTCCACTAATCGGTGGAGGATGATGACCGGTCCCATTCCTTTTG

genome         CCCCAACTTGTCAGGACTCAAGAGGCTCCCCTCCTCCCTCGCCGCGGAACACGCGCGCCG
sequence       CCTCAATTTGTTAGGACTCAAGAGGCTTCCTTTTTTCCTTGTTGTGGAATATGTGCGTCG

genome         TCGTCGCCAGCCCGGGTTCATTTCTCCGTTCCTTGGTAAGCAAGTCTAGGTTT    -355
sequence       TTGTTGCTAGTTTGGGTTTATTTTTTCGTTCTTTGGTAAGCAAGTCTAGGTTT
```

Supplementary Figure S2. Distribution of CG sites in the *Sdh3-2* gene promoter and the methyl status of cytosine in scutella during germination of maize seeds. Thymine, formed from cytosine during bisulfite conversion of DNA, is underlined. "Genome" is the genomic DNA sequence of the promoter of the gene under study, taken from the NCBI database. "Sequence" is the sequence of the amplicon obtained using primers for bisulfite sequencing to the corresponding gene promoter. The highlighted regions on the sequence are the analyzed cytosine methylation sites.

Sdh4

Day 1

```
genome                -411  AAGAACAGCATAAGTACCTCCCTTCTGGTCGGCGCCGTGTT
sequence                    AAATACAGTATAAGTATTTCTTTTCTGGTCGGCGCTGTGTT

genome    CCATCAAGTATACATGTTCCACAAGGGGATTCTTCTGGACTACGTGCACCATGATATCAC
sequence  CTATTAAGTATACATGTTCCACAAGGGGGTTTATTGGATAACGTGCACCATGATATTAG

genome    CAGAAAGTGGGCTTTGATTTACTTCAAGCTGCTTTTGCTGGTGATGGCGAAGGACACCAT
sequence  CAGAAAGTGGGTATTGATTTACTTCAAGTTGCTTTTGTTGGTGATGGCGAAGGGTGTCAT

genome    CATGTACTTCAATCTGTTCTAAGCTCACCTTCTCAGTTTCTCAAATATCCAGCAATGCAT
sequence  CATGTACTTCAATCTGTTTTAAGCTCATTTTCTCAGTTTCTCAAATATCTAGCAATGCAT

genome    CCGTCTCTGCACTCGGTAGAATAATGATGATGGATGAA    -153
sequence  TTGTCTCTGCACTCGGTAGAATAATGATGAAGGATGAA
```

Day 4

```
genome                -411  AAGAACAGCATAAGTACCTCCCTTCTGGTCGGCGCCGTGTT
sequence                    GAGAACAGCATAAGTATGTTCTTATTGGACGGGGCCGTGTT

genome    CCATCAAGTATACATGTTCCACAAGGGGATTCTTCTGGACTACGTGCACCATGATATCAC
sequence  TCATTAAGTATACATGTTTCATAAGGAGATTCTTCTGGACTACGTGCATTATGATATTAC

genome    CAGAAAGTGGGCTTTGATTTACTTCAAGCTGCTTTTGCTGGTGATGGCGAAGGACACCAT
sequence  CAGAAAGTGGGCTTTGATTTATTTCAAGCTGGTTTTGTTGGGTATGGGGAAGGACACTAT

genome    CATGTACTTCAATCTGTTCTAAGCTCACCTTCTCAGTTTCTCAAATATCCAGCAATGCAT
sequence  TATGTACTCCAATTTGTTTTAAGCTCATCTTCTCAGTTTCTCAAATATCCAGCAATGCAT

genome    CCGTCTCTGCACTCGGTAGAATAATGATGATGGATGAA   -153
sequence  TTGTCTTTGCACTTGGTAGAATAATGATGATGGATGAA
```

Day 8

```
genome                -411  AAGAACAGCATAAGTACCTCCCTTCTGGTCGGCGCCGTGTT
sequence                    GAGAACAGCATAAGTATTTCCCTTTTGATCGGCGCTGGGTT

genome    CCATCAAGTATACATGTTCCACAAGGGGATTCTTCTGGACTACGTGCACCATGATATCAC
sequence  TTGGAAAGTATATATGTTCTACAAGGGGATTCTTCTGGATTACGTGGACCATGGTATCAC

genome    CAGAAAGTGGGCTTTGATTTACTTCAAGCTGCTTTTGCTGGTGATGGCGAAGGACACCAT
sequence  CAGAAAAAGGGTTTTGATTTACTTCAAGCTGTTTTTGCTGGTGATGGTGAAGGACACTAT

genome    CATGTACTTCAATCTGTTCTAAGCTCACCTTCTCAGTTTCTCAAATATCCAGCAATGCAT
sequence  TATGTACTTTAATCTGTTCTAAGCTCAGTTTCTTAGTTTTTCAAATATTTAGCAATGCAT

genome    CCGTCTCTGCACTCGGTAGAATAATGATGATGGATGAA   -153
sequence  TT GTCTTTGCATTTGGTAGAATAATGATGATGGATGAA
```

Supplementary Figure S3. Distribution of CG sites in the *Sdh4* gene promoter and the methyl status of cytosine in scutella during germination of maize seeds. Thymine, formed from cytosine during bisulfite conversion of DNA, is underlined. “Genome” is the genomic DNA sequence of the promoter of the gene under study, taken from the NCBI database. “Sequence” is the sequence of the amplicon obtained using primers for bisulfite sequencing to the corresponding gene promoter. The highlighted regions on the sequence are the analyzed cytosine methylation sites.
